# CBX6 overexpression contributes to tumor progression and is predictive of a poor prognosis in hepatocellular carcinoma

**DOI:** 10.18632/oncotarget.14770

**Published:** 2017-01-20

**Authors:** Hao Zheng, Wei-hua Jiang, Tao Tian, Hai-song Tan, Ying Chen, Guang-lei Qiao, Jun Han, Sheng-yu Huang, Yuan Yang, Shuai Li, Zhen-guang Wang, Rong Gao, Hao Ren, Hao Xing, Jun-sheng Ni, Lin-Hui Wang, Li-jun Ma, Wei-ping Zhou

**Affiliations:** ^1^ The Third Department of Hepatic Surgery, Eastern Hepatobiliary Surgery Hospital, Second Military Medical University, Shanghai 200433, China; ^2^ Department of Oncology, Shanghai Tongren Hospital, Shanghai Jiaotong University, Shanghai 200336, China; ^3^ Department of Urology, Changzheng Hospital, Second Military Medical University, Shanghai 200003, China; ^4^ Department of Computer Science, Rensselaer Polytechnic Institute, Troy, NY, 12180, United States of America; ^5^ Department of Microbiology, Shanghai Key Laboratory of Medical Biodefense, Second Military Medical University, Shanghai 200433, China; ^6^ The Fourth Department of Hepatic Surgery, Eastern Hepatobiliary Surgery Hospital, Second Military Medical University, Shanghai 200433, China

**Keywords:** CBX6, S100A9, hepatocellular carcinoma, proliferation, biomarker

## Abstract

Aberrant chromobox (CBX) family protein expression has been reported in a variety of human malignancies. However, the role of CBX6 in hepatocellular carcinoma (HCC) progression and patient prognosis remains unknown. In this study, we found that CBX6 was frequently up-regulated in HCC clinical samples and HCC cell lines and that CBX6 expression was significantly correlated with larger tumor sizes (≥ 5 cm, *p* = 0.011) and multiple tumors (*n* ≥ 2, *p* = 0.018). Survival analyses indicated that patients with higher CBX6 expression levels had significantly shorter recurrence-free survival (RFS) and overall survival (OS) than patients with lower CBX6 expression levels, and multivariate analyses confirmed that increased CBX6 expression was an independent unfavorable prognostic factor for HCC patients. Functional study demonstrated that CBX6 profoundly promoted HCC cell growth both *in vitro* and *in vivo*, and mechanistic investigation revealed that the S100A9/NF-κB/MAPK pathway was essential for mediating CBX6 function. In conclusion, our results represent the first evidence that CBX6 contributes to tumor progression and indicate that the protein may serve as a novel prognostic biomarker for HCC and as a therapeutic target in the treatment of the disease.

## INTRODUCTION

Primary liver cancer is the fifth-most common cancer worldwide and the third-most common cause of cancer mortality. Hepatocellular carcinoma (HCC) accounts for nearly 90% of primary liver cancers [1, 2]. HCC always develops in the background of chronic hepatitis or cirrhosis. Inflammatory cells invade the liver as a result of the destruction of large numbers of hepatocytes, leading to the deposition of connective tissue in chronic liver disease [3–5]. Although multiple tumor suppressor genes and oncogenes that participate in HCC development and progression have been identified [6, 7], our knowledge of the cellular and molecular pathways underlying HCC progression remains limited.

The chromobox (CBX) protein family comprises chromodomain-containing proteins involved in regulating gene expression, cell self-renewal and differentiation [8–11]. The overall structures of CBX proteins are similar, and these proteins direct the localization of their respective complexes through specific recognition of distinct but sequence-related repressive marks on histone H3 trimethylated at Lys-9 (H3K9me3) and Lys-27 (H3K27me3) [12–16]. The detailed functions of each protein remain unclear; however, these proteins play important roles in disease progression. CBX1, 3, and 5 are associated with gene repression in heterochromatin, and CBX7 and 8 are key regulators of developmental genes targeted by H3K27me3. CBX2, 4, and 6 also perform this function but have weaker effects than their counterparts [9–11, 16]. CBX6 shows a high degree of conservation, but its chromodomains display significant differences from those of other proteins with respect to their histone peptide binding preferences. Previous studies have reported that CBX6 may be a reader of different, as-yet undetermined trimethyllysine sites and may be capable of binding to RNA [9, 15, 17, 18]. However, the role of CBX6 in HCC development and progression has not been explored.

In the present study, we demonstrated that CBX6 expression is increased in HCC cells and tumor tissues and that increased CBX6 expression is associated with poor outcomes in a large HCC cohort. Additionally, the results of our univariate and multivariate analyses indicated that CBX6 was an independent prognostic factor for poor survival and HCC recurrence after surgery. Moreover, CBX6 up-regulation dramatically promoted HCC proliferation both *in vitro* and *in vivo*, while CBX6 down-regulation dramatically attenuated HCC proliferation both *in vitro* and *in vivo*. Regarding the mechanism underlying the involvement of CBX6 in HCC, gene microarray analysis of a CBX6-knockdown cell line revealed that S100A9 expression was low, suggesting that CBX6 plays an important role in HCC progression through S100A9/NF-κB/MAPK signaling and may be useful as a future HCC therapy.

## RESULTS

### CBX6 expression is frequently increased in human HCC tissues

**Figure 1:**
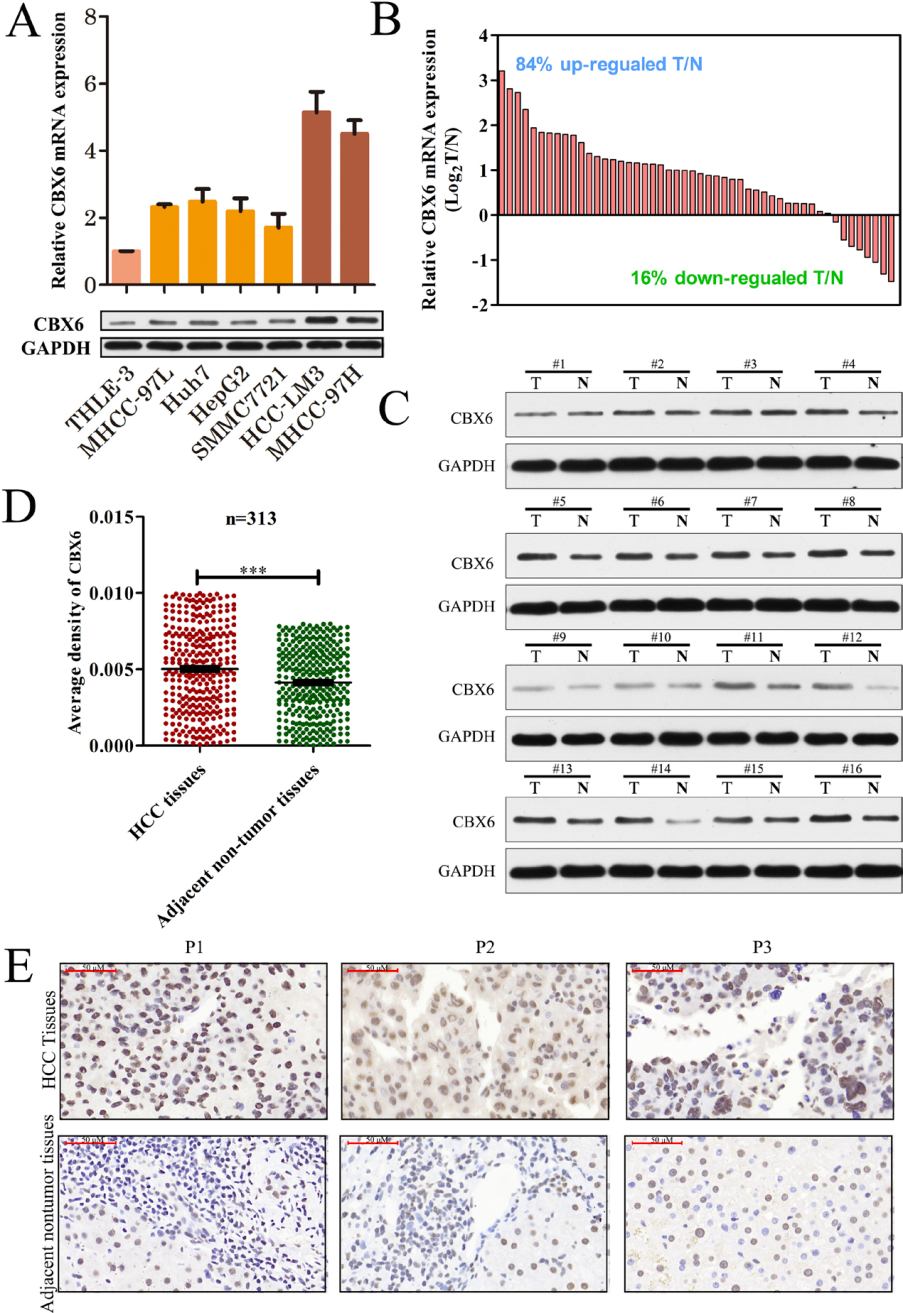
CBX6 expression is frequently increased in human HCC tissues (**A**) Relative CBX6 mRNA and protein expression levels in the THLE-3 and HCC cell lines were determined by real-time qPCR and western blot assay. The gene expression results were normalized to GAPDH, which served as an internal control. (**B**) CBX6 mRNA expression levels in 50 pairs of HCC tissues and matched adjacent non-tumor tissues were evaluated by qPCR. (**C**)Western blot analysis of CBX6 protein levels in tumor tissues and paired adjacent non-tumor tissues from 16 patients with HCC. (**D**)Relative IHC staining for CBX6 expression in HCC tissue samples and paired adjacent non-tumor tissue samples (*n* = 313).CBX6 expression was significantly increased in the tumor tissue samples compared with the corresponding adjacent non-tumor tissue samples. (**E**)Representative IHC image of tumor tissues and peri-tumor tissues (*p < 0.05;**p < 0.01;***p < 0.001).

To determine the significance of CBX6 expression in HCC, we first detected CBX6 mRNA and protein expression levels in a normal human liver cell line (THLE-3) and several human HCC cell lines (MHCC-97L, Huh7, HepG2, SMMC7721, HCCLM3, and MHCC-97H). Real-time quantitative polymerase chain reaction (qRT-PCR) and western blot analyses revealed that CBX6 mRNA and protein expression levels were both markedly increased in all six HCC cell lines compared to the THLE-3 cell line (Figure 1A).Then, we detected CBX6 mRNA expression in 50 pairs of primary HCC tissue samples and corresponding adjacent nontumor tissue samples. Our qPCR results showed that relative CBX6 mRNA expression levels were significantly higher in the tumor tissue samples than the adjacent non-tumor tissue samples, as 84% (42/50) of the HCC tissue specimens showed a higher CBX6 mRNA expression level than their matched non-tumor counterparts (Figure 1B). Similar results were observed in the western blot analysis (Figure 1C). To determine the protein expression patterns of CBX6 in the HCC clinical samples, we next performed immunohistochemistry (IHC) forCBX6 expression using HCC tissue microarrays (TMAs) containing 313 HCC samples and paired peri-tumor samples. As shown in Figure 1D, the staining intensity of the CBX6 protein in the tumor group was stronger than that in the peri-tumor group. Representative IHC staining results are shown in Figure 1E. We also detected the expression of other CBX family proteins in 50 pairs of HCC tissues and adjacent non-tumor tissues, and no up-regulations of CBX1-5,CBX 7, or CBX 8 expression were observed in the HCC tissue samples (Supplementary Figure 1A–1G). Our results revealed that CBX6 expression levels were frequently increased in HCC, implying that the protein plays an oncogenic role in the disease.

### Association between CBX6expression and HCC patient clinicopathologic characteristics

To further investigate the clinical significance of CBX6 expression in HCC development and progression, we divided all 313 HCC patients into the following 2 groups based on their overall CBX6 expression levels: a high CBX6 expression group (*n* = 157) and a low CBX6 expression group (*n* = 156). We found that high CBX6expression was more frequent in HCC patients with larger tumor sizes (≥ 5 cm, *p* = 0.011) and multiple tumors (*n* ≥ 2, *p* = 0.018) (Table 1) than in HCC patients with smaller tumor sizes and single tumors.

**Table 1:** Clinical characteristics of 313 HCC patients according to their CBX6 expression levels

Feature	CBX6		*χ*^2^	*p*-value
	High	Low		
All cases				
Age, y			0.026	0.872
≥ 55	58	59		
< 55	99	97		
Gender			0.000	0.984
Male	139	138		
Female	18	18		
Hepatitis B ag			0.739	0.390
Positive	139	133		
Negative	18	23		
Hepatitis E ag			0.643	0.422
Positive	36	30		
Negative	121	126		
AFP, μg/L			0.006	0.941
Positive	99	99		
Negative	58	57		
Tumor size, cm			6.512	0.011
≥ 5	98	75		
< 5	59	81		
Tumor number			5.608	0.018
Single	117	133		
Multiple	40	23		
Vascular invasion			0.729	0.393
Present	98	90		
Absent	59	66		
Tumor differentiation			1.145	0.285
I–II	20	14		
III–IV	137	142		

Values in red are statistically significant (*p* < 0.05).

AFP, alpha-fetoprotein; HBsAg, hepatitis B surface antigen; HBeAg, hepatitis Be antigen; negative: < 20 ng/ml, positive: ≥ 20 ng/ml.

^a^Patients whose tumor tissue immunohistochemistry score was > 1 constituted the high-expression group, and the remaining patients constituted the low-expression group.

### Relationship between CBX6 expression and HCC patient prognosis

**Figure 2:**
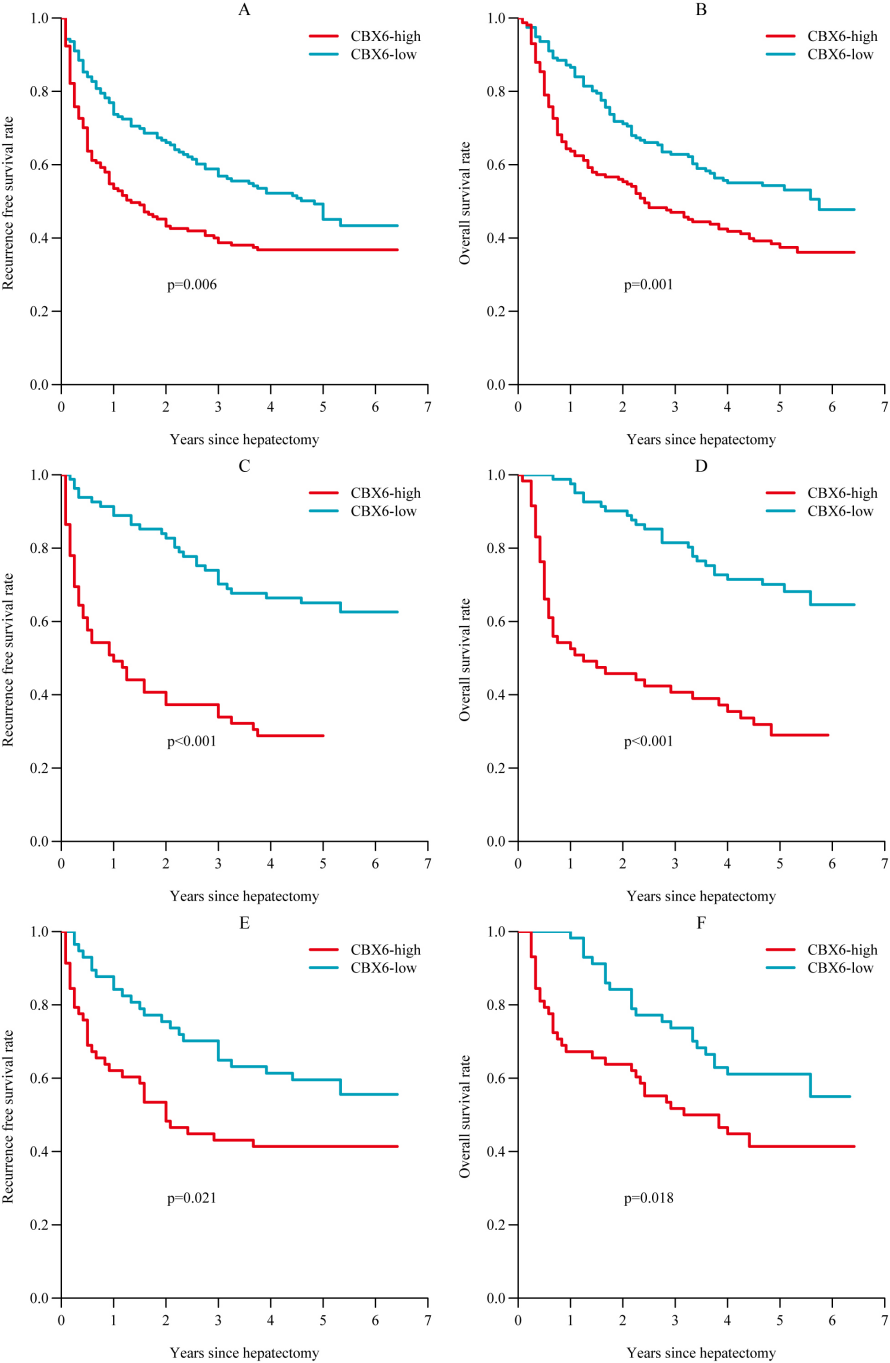
Relationship between CBX6 expression and HCC patient prognosis (**A** and **B**) Postoperative RFS and OS among all HCC patients. (**C** and **D**) Postoperative RFS and OS among HCC patients with a tumor size < 5 cm. (**E** and **F**) Postoperative RFS and OS among HCC patients with an AFP < 20 ng/ul.Statistical significance was assessed by two-sided log-rank tests (*p < 0.05;**p < 0.01;***p < 0.001).

We analyzed the association between CBX6 protein levels and HCC patient prognosis after hepatectomy. We found that the high CBX6expression group had significantly poorer recurrence-free survival (RFS) (*p* = 0.006, Figure 2A) and poorer overall survival (OS) (*p* = 0.001, Figure 2B) than the low CBX6 expression group. Subgroup analysis revealed that among patients with a tumor size < 5 cm (140 patients), differences in RFS and OS still existed between the high and low CBX6 expression groups (*p <* 0.001; respectively, Figure 2C and 2D). Further analysis revealed that among AFCP-negative patients (115 patients), the high CBX6 expression group had significantly poorer RFS (*p* = 0.021, Figure 2E) and poorer OS than the low CBX6 expression group (*p* = 0.018, Figure 2F). Univariate analysis showed that of the clinicopathological characteristics analyzed herein, CBX6 expression levels, tumor size, vascular invasion, and the presence of AFP, HBsAg, or HBeAg were correlated with RFS, and CBX6 expression levels, tumor size, the presence of HBsAg, and the presence of HBeAg were correlated with OS (Supplementary Table 1). Furthermore, multivariate Cox regression analysis showed that CBX6 expression levels, vascular invasion, the presence of HBeAg, and the presence of HBsAg were independent risk factors for HCC recurrence and that CBX6 expression levels, the presence of HBeAg, and the presence of HBsAg were independent risk factors for OS in HCC patients (Table 2). Taken together, these data indicate that CBX6expression levels can be used as an independent factor for predicting the prognosis of HCC.

**Table 2:** Multivariable analysis of recurrence free survival (RFS) and overall survival (OS) in patients

Variable	RFS				OS			
	*P*	HR	95% CI		*P*	HR	95% CI	
**HBsAg**, positive vs. negative	0.020	1.839	1.099	2.318	0.019	1.927	1.114	3.332
**HBeAg**, positive vs. negative	0.003	1.662	1.192	2.318	0.003	1.656	1.185	2.314
**Vascular invasion**, present vs. absent	0.004	1.580	1.160	2.152	–	–	–	–
**CBX6 expression**, high vs. low	0.018	1.423	1.061	1.908	0.003	1.591	1.175	2.153

Abbreviation: HBsAg, hepatitis B surface antigen; HBeAg, hepatitis B e antigen.

### CBX6 promotes HCC cell proliferation and HCC cell clonogenicity *in vitro*

**Figure 3:**
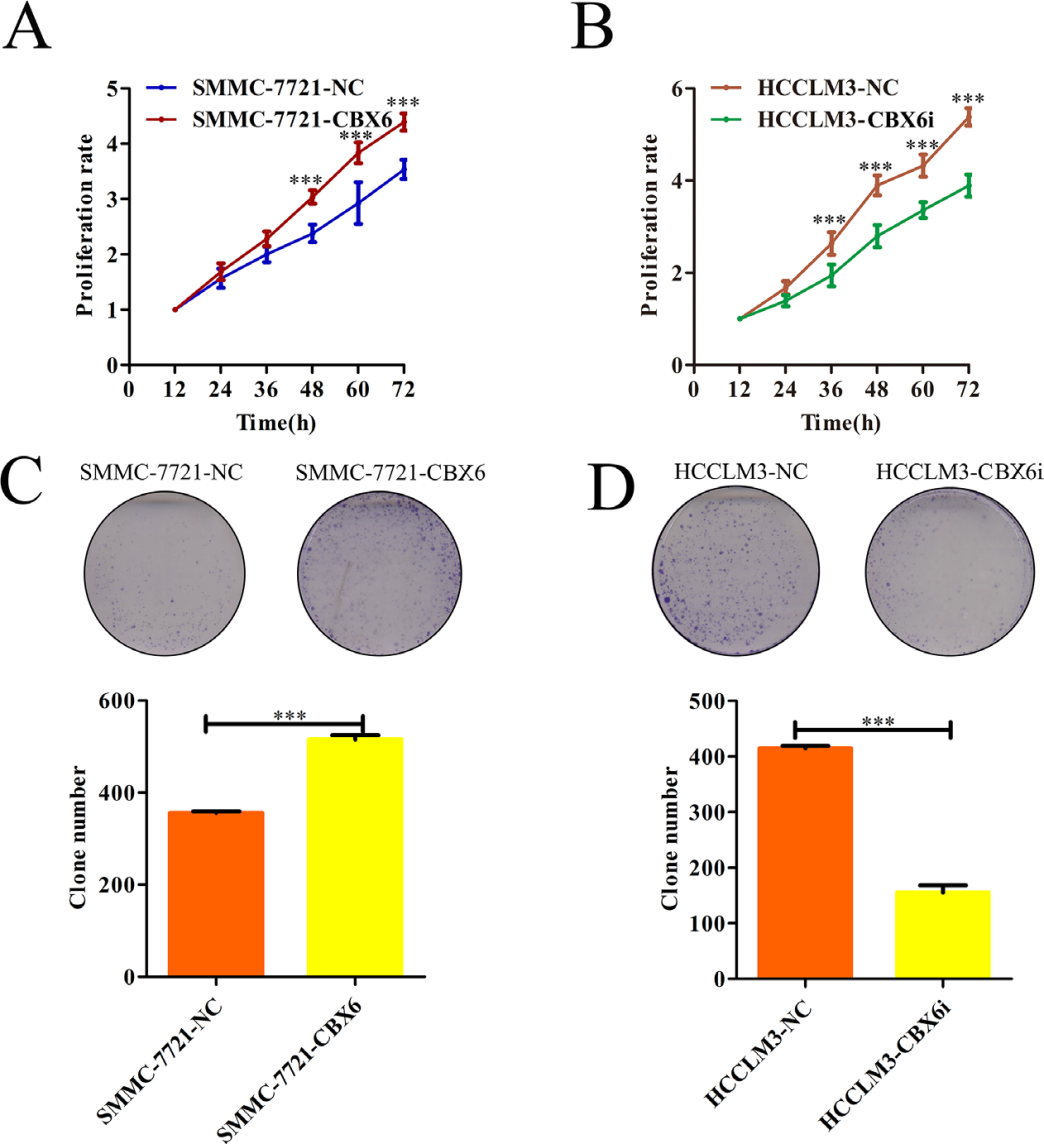
CBX6 promotes HCC cell proliferation and HCC cell clonogenicity *in vitro* (**A** and **B**) Cell proliferation was determined by CCK-8 assay, and growth curves were generated by reading the absorbance values at different time points. Growth curves of (A) CXB6-overexpression cells and (B) CBX6-knockdown cells and their respective controls are shown. (**C** and **D**) Representative results of the colony formation assays of (C) CBX6-overexpression cells, (D) CBX6 knockdown cells and their respective controls. *p < 0.05; **p < 0.01; ***p < 0.001 compared with the respective controls. Significance was determined from three independent experiments and assessed by Student's *t*-test. Data are shown as the mean ± SD.

Given the significant correlation between CBX6expression levels and HCC clinical invasiveness, it is likely that CBX6 plays a positive role in cancer progression. To investigate the biological significance of CBX6 *in vitro*, we examined the effects of CBX6 loss-of-function and gain-of-function on cell phenotype. SMMC7721cells exhibited lower CBX6 expression and were thus selected to generate stable CBX6 over-expression cells (which we named SMMC-7721-CBX6), and HCCLM3 cells exhibited higher CBX6 expression and were thus selected to generate stable CBX6-knockdown cells (which we named HCCLM3-CBX6i).CBX6overexpression or knockdown efficiency was confirmed by qRT-PCR and western blotting (Supplementary Figure 2).Cell Counting Kit-8 (CCK8) was utilized to assess HCC cell proliferation *in vitro*. As shown in Figure 3A, CBX6 overexpression promoted SMMC-7721 cell proliferation, and the difference in cell growth between the knockdown and control cell lines continued to increase at subsequent time points. Conversely, HCC-LM3 cells with CBX6 knockdown displayed notable reductions in their proliferation rate compared with their corresponding control cells (Figure 3B). To evaluate the long-term effects of CBX6 on cell proliferation, we performed colony-formation assays. As shown in Figure 3C and 3D, significantly more tumor colonies had formed in the CBX6-overexpression group than in the CBX6-knockdowngroup on day 14. We next examined the anti-apoptotic effects of CBX6 in HCC cells via flow cytometric analysis. Surprisingly, no significant differences in apoptosis were noted between the stable CBX6-overexpression cell line and its control cell line or between the CBX6-knockdown cell line and its control cell line following treatment with apoptosis-inducers A (Apopida) and B (Apobid) for 6 h (Supplementary Figure 3A). Apoptosis-associated protein expression levels in the SMMC-7721-CBX6 and HCC-LM3-CBX6i cell lines and their respective control cell lines were also evaluated by western blotting. Similar to the above results, no differences in apoptosis-associated protein levels were observed between the above cells and their respective control cells (Supplementary Figure 3B). These results indicate that CBX6has no anti-apoptotic effects in HCC cells. Taken together, our results indicate that CBX6 promotes HCC cell proliferation and HCC cell clonogenicity *in vitro*.

### CBX6 promotes HCC tumor growth *in vivo*

**Figure 4:**
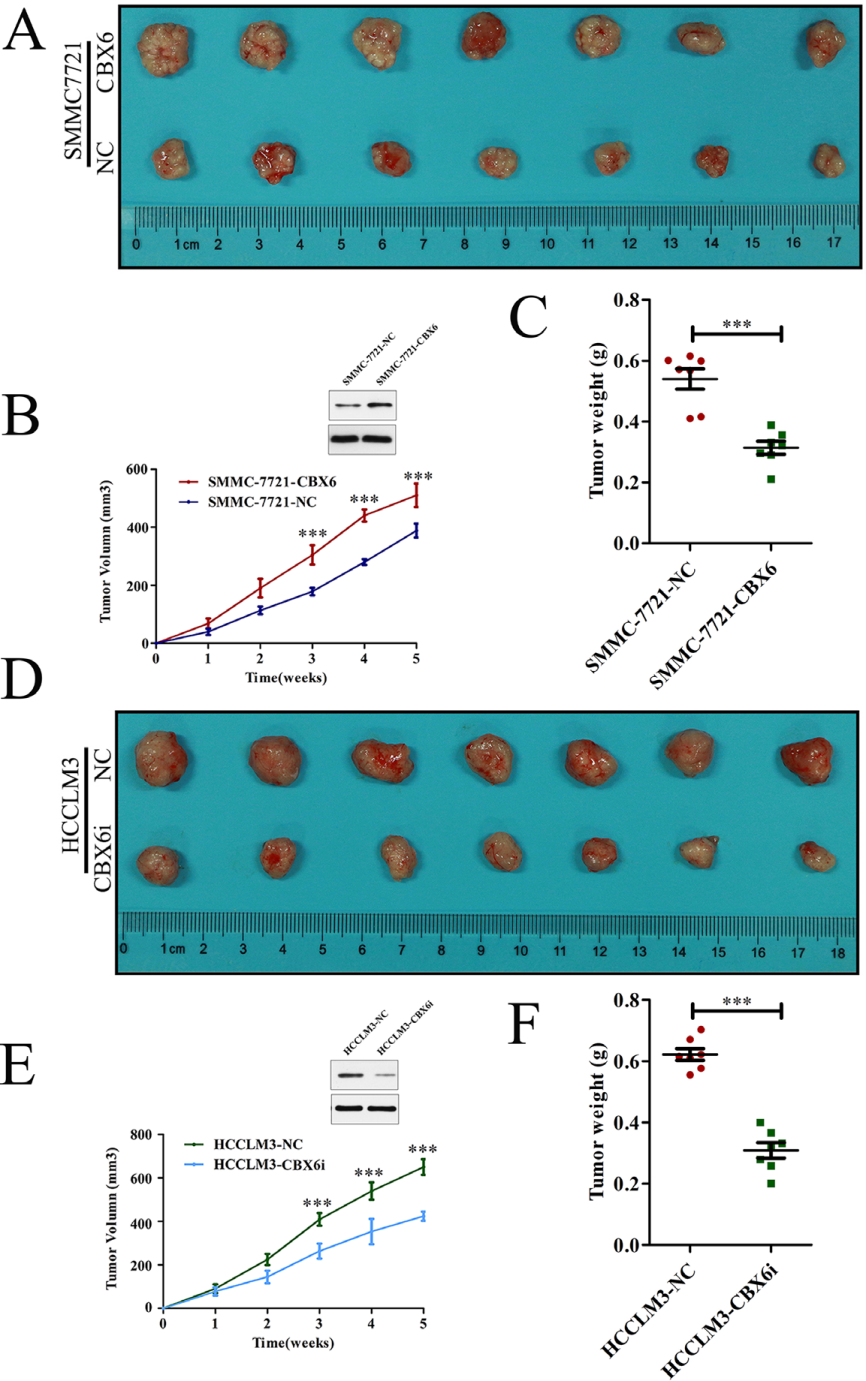
CBX6 promotes HCC tumor growth *in vivo* Mice received subcutaneous injections of 1 × 10^7^ (**A**–**C**) SMMC-7721-CBX6 and SMMC-772-NC cells and (**D**–**F**) HCCLM3-CBX6i and HCCLM3-NC cells. (A and D) The mice and their tumors were evaluated five weeks post-injection, and representative images are shown. (B and E) Tumor growth curves and (C and F) tumor weights for the five-week study period are shown. Data are shown as the mean ± SEM. Statistically significant differences were identified by Student's *t*-tests. *p < 0.05; **p < 0.01; ***p < 0.001 compared with the appropriate controls.

To determine the effects of CBX6 on tumourigenesis *in vivo*, we subcutaneously injected SMMC-7721-NC cells or SMMC-7721-CBX6cells into nude mice for xenotransplantation. The results of this experiment showed that CBX6 overexpression evidently promotedtumor growth. As shown in Figure 4A, mice injected with SMMC-7721-CBX6 cells showed significantly increased tumor growth compared with mice injected with SMMC-7721-NC cells. The results of our tumor volume and mass measurements indicated thatCBX6 overexpression significantly increased overall tumor growth (Figure 4B and 4C). We performed similar assessments of xenograft tumor formation in nude mice using CBX6 knockdown cells and the appropriate control cells. As shown in Figure 4D–4F, CBX6 knockdown tumors (HCCLM3- CBX6i) showed significantly decreased tumor growth compared with control tumors (HCCLM3-NC), based on our measurements of tumor volume and weight. We also measured the levels of two cellular proliferation antigens, Ki67 and PCNA, in tumor tissues from the xenografts via IHC and found that the expression levels of both of these antigens were significantly stronger in theSMMC-7721-CBX6cell xenografts than in theSMMC-7721-NC cell xenografts (Supplementary Figure 4A).In contrast, the expression levels of Ki67 and PCNA were significantly lower in the HCCM3-CBX6i cell xenografts than in the HCCLM3-NCcell xenografts (Supplementary Figure 4B). These results revealed that CBX6 was capable of promoting tumor growth *in vivo* and acted as an oncogene in HCC.

### CBX6 increased S100A9 expression in HCC

**Figure 5:**
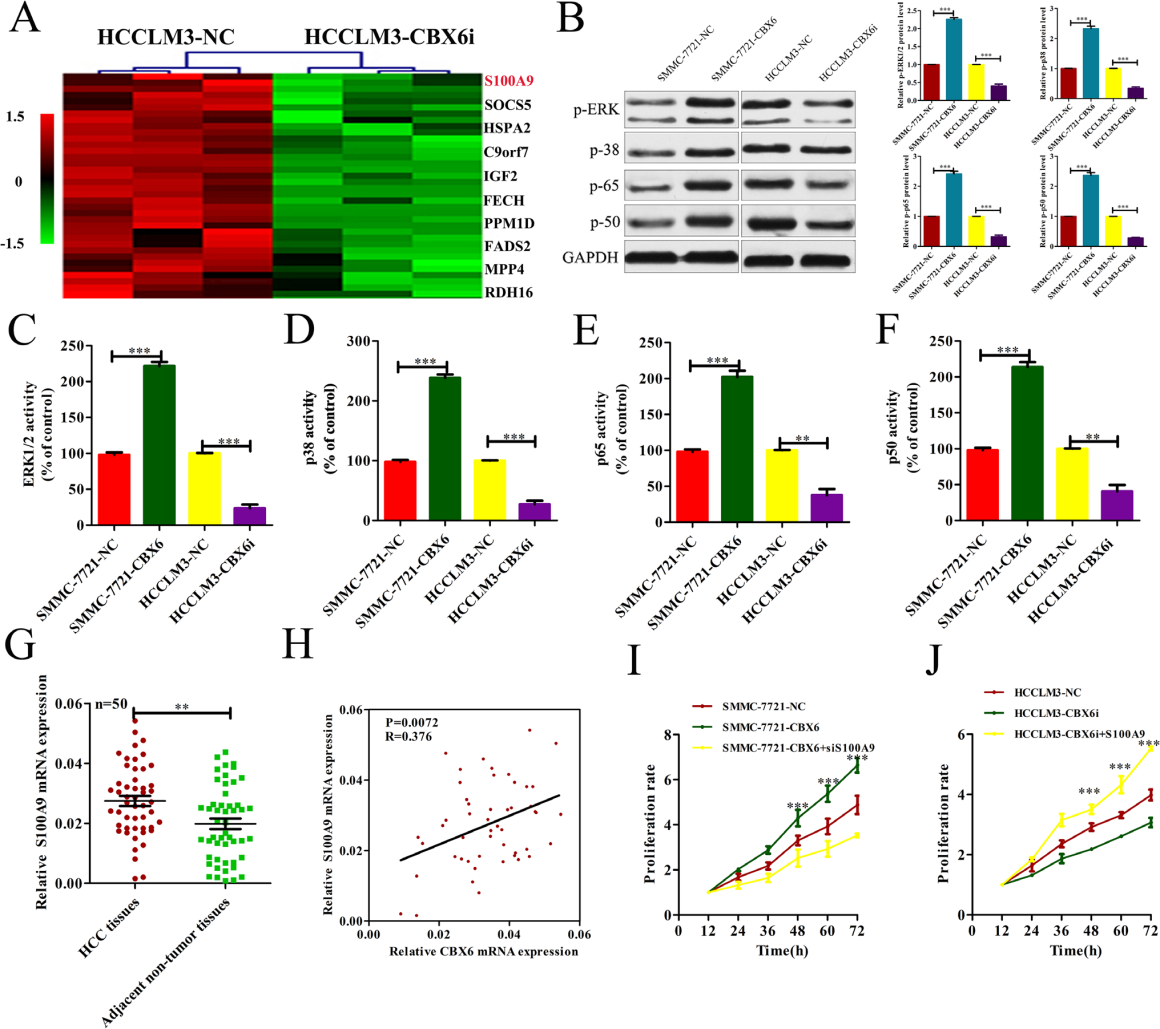
CBX6 increased S100A9 expression, and S100A9 plays a critical role in mediating CBX6 function in HCC (**A**) Genes differentially expressed between HCCLM3-CBX6i cells and control cells. The top 10 down-regulated genes are listed on the right. (**B**) ERK1/2 MAPK, p38 MAPK, p50 NF-κB and p65 NF-κB phosphorylation levels in each group were detected by western blotting. (**C**–**F**) ERK1/2 MAPK, p38 MAPK, p50 NF-κB and p65 NF-κB activity levels in each group were quantified by spectrophotometricassay. (**G**) S100A9 mRNA levels in primary HCC tissue samples and adjacentnontumor tissuesamples from the same set of patients as those described in Figure 1B were measured by qRT-PCR. (**H**) The correlation between CBX6 transcript levels and S100A9 mRNA levels was measured in the same set of HCC tissues as those described in (G). (**I**) CCK8 assay for SMMC7721 cells overexpressing CBX6 and SMMC7721 cells overexpressing CBX6 that were transfected with a S100A9-knockdown vector or a control vector. (**J**) CCK8 assay for HCCLM3 cells treated with CBX6 knockdown and HCCLM3 cells treated with CBX6 knockdown that were transfected with an S100A9 vector or a control vector.*p < 0.05; **p < 0.01; ***p < 0.001.

To identify the genes that are potentially involved in CBX6-mediatedcell proliferation, we constructed gene microarrays. The gene expression data were obtained from three independent competitive hybridizations comparing PON3-knockdown HCCLM3 cells (HCCLM3-CBX6i) with control cells (HCCLM3-NC). A corrected *p*-value < 0.05 and an absolute fold change > 1.2 were the criteria used for identifying differentially expressed genes. A total of 240 genes were found to be differentially expressed. Of these, 115 genes were down-regulated, and 125 genes were up-regulated. We found that the expression of S100A9, one of the top ten down-regulated genes in HCCLM3-CBX6i cells, was dramatically induced by CBX6 (Figure 5A). Given that S100A9 functions in cell proliferation [19], we surmised that CBX6may promote proliferation by increasing S100A9 expression. The results of previous studies indicated that S100A9 regulated MAPK signaling and NF-Κb signaling [20]; therefore, we tested the phosphorylation of the MAPK and NF-κB signaling pathways by western blot analysis and enzyme activity assay. Western blot analysis revealed that the protein expression levels of phospho-ERK1/2 MAPK, phospho-p38 MAPK, phospho-p50 NF-κB and phospho-p65 NF-κB in the SMMC-7721-CBX6 cell line were higher than those of the same proteins in the SMMC-7721-NCcell line and that the protein expression levels of phospho-ERK1/2 MAPK, phospho-p38 MAPK, phospho-p50 NF-κB and phospho-p65 NF-κB in the HCCLM3-CBX6i cell line were lower than those of the same proteins in the HCCLM3-NC cell line (Figure 5B). Enzyme activity assay confirmed the above results (Figure 5C–5F). To confirm that S100A9 is regulated by CBX6 in human HCC tissues, we measured S100A9 mRNA levels in 50 pairs of HCC tissues and matched nontumor tissues, as shown in Figure 1B,and found that S100A9 mRNA levels were significantly higher in the HCC tissue samples than in the primary nontumor tissue samples (Figure 5G). Importantly, S100A9 mRNA levels were correlated withCBX6 transcript levels in the HCC tissue samples (Figure 5H).These clinical data indicate that S100A9 may be associated with HCC cell metastasis and were consistent with our hypothesis regarding the role of CBX6 in S100A9 regulation.

### S100A9 plays a critical role in mediating CBX6 function

After confirming that CBX6 can regulate S100A9 expression in HCC, we explored whether S100A9 can mediate the biological function of CBX6 in HCC cells further. We found that S100A9 lentivirus transection significantly increased S100A9 expression levels in HCCLM3-CBX6i cells (*p <* 0.01, Supplementary Figure 5A), while S100A9 siRNA significantly reduced S100A9 expression levels in SMMC-772-CBX6 cells (*p <* 0.01, Supplemental Figure 5B).Functionally, S100A9 knockdown abrogated the positive effects ofCBX6on SMMC-7721-CBX6 cell proliferation (Figure 5I).However, S100A9 overexpression abrogated the inhibitory effects of CBX6 knockdown on HCCLM3-CBX6i cell proliferation (Figure 5J). These results suggest that S100A9 is not only a downstream target of CBX6 but also a functional mediator of the effects of CBX6 in HCC.

## DISCUSSION

The histopathological and molecular features that lead to HCC initiation and progression are still poorly understood [21]. Viral infection, metabolic alterations leading to chronic inflammation and epigenetic and genetic changes cooperate in cancer development [22].Hepatocarcinogenesis is a complex process characterized by increases in the expression of several factors that influence the survival of cancer cells by suppressing apoptosis and regulating cell cycle [23–25]. Increases in the incidence of HCC have led to the performance of intense research intended to elucidate the detailed mechanisms underlying HCC development.

In the present study, we showed that CBX6 expression in HCC tissues was significantly up-regulated compared with that in adjacent normal liver tissues. The results of our IHC analysis of TMA slides from a cohort of 313 randomly selected HCC patients and our Kaplan-Meier analysis showed that up-regulated CBX6 expression was predictive of poor HCC patient survival. These results suggest that CBX6 may play an important role in HCC progression. The results of a previous study indicated that patients with a tumor size < 5 cm who are AFP negative are generally considered to have a better prognosis than patients with a tumor size > 5 cm who are AFP positive [26–29]; however, many of these patients still experience early disease recurrence and poor OS. Therefore, a precise biomarker for HCC is needed to predict the prognoses of patients with a smaller tumor size who are AFP negative. In our study, we found that within these populations, the high-expression group still had significantly poorer RFS than the low-expression group. Thus, the findings of the present study suggest that measuring CBX6protein levels may enable clinicians to identify patients with early stage disease who face a worse prognosis than other patients with early stage disease.CBX6 may thus have prognostic value because it may allow clinicians to distinguish between early stage patients with a high risk of recurrence and early stage patients with a low risk of recurrence. In the former group of patients, close follow-up and appropriate adjuvant therapies should be recommended to prolong survival.

By analyzing the association between CBX6 protein levels and clinicopathological characteristics using TMA analysis, we found that high CBX6expression was more frequent in HCC patients with a larger tumor size (≥ 5 cm, *p* = 0.011) and multiple tumors (*n* ≥ 2, *p* = 0.018) than in patients with a smaller tumor size and single tumors, indicating that CBX6expression is closely correlated with tumor progression. We next explored the possible function of CBX6 in HCC progression. The results of this experiment showed that CBX6 promoted HCC cell proliferation *in vitro* and *in vivo*. Taken together, our data indicate that CBX6 plays an important role in HCC progression and suggest that CBX6may be useful as a novel biomarker that can predict HCC prognosis. Our results also indicate that CBX6 may be a therapeutic target in the treatment of HCC; however, the molecular mechanisms underlying the involvement of CBX6 in HCC proliferation remain to be elucidated.

To identify genes that are potentially involved in CBX6-mediated cell proliferation, we constructed gene microarrays. We found that the expression of S100A9, one of the top ten down-regulated genes, was significantly decreased. S100A9 belongs to the S100 gene cluster and was originally discovered inhuman granulocytes and macrophages [30–33].S100A9 has been shown to be associated with myelomonocytic cell differentiation [34], andit is well known that S100A9 deregulation is associated with several common human malignancies and induces neutrophil adhesion to fibronectin [35, 36].

In a previous study, S100A9 was found to mediate proliferative signals and enhance the MAPK or NF-κB signaling pathway [37]. Aberrant activation of both the MAPK and the NF-κB signaling pathway has critical effects on tumor growth [38–41], according to the results previous studies [42]. We observed that CBX6suppression caused decreases in ERK1/2 phosphorylation and MAPK-p38, NF-κB-p50, and NF-κB-p65 phosphorylation and thatCBX6overexpression increased ERK1/2 phosphorylation and MAPK-p38,NF-κB-p50, and NF-kB-p65 phosphorylation, indicating that CBX6may mediate tumor progression in HCC cells by promoting cellular proliferation, which is linked to the MAPK and NF-κB signaling pathways. We also found that knocking downS100A9using siRNA decreased HCC cell proliferation and reversed the HCC cell proliferation enhancements induced by CBX6 and that S100A9 overexpression increased HCC proliferation and reversed the decrease in proliferation induced by CBX6 knockdown. All of these results suggest that CBX6 promotescell proliferation, partly through S100A9 up-regulation. However, as described above, as information regarding the precise mechanism by which CBX6 increases S100A9 expression is lacking, the relationship between CBX6 and S100A9 is still unclear and requires further investigation.

In conclusion, we investigated the expression of CBX6 and its value as a prognostic biomarker for HCC. We also explored the biological function of CBX6 in HCC and observed that CBX6 up-regulation is a predictor of high risks of metastasis and recurrence in HCC patients. High-risk patients should be closely monitored and should receive appropriate adjuvant therapies so that their prognoses can be improved. We also found that CBX6 promoted HCC proliferation by elevating S100A9 expression. These findings suggest that CBX6 plays an important role in HCC development and progression and may be a useful therapeutic target in the treatment of the disease.

## MATERIALS AND METHODS

### Patient characteristics and tissue specimens

All the patients enrolled in our study were infected with HBV. We obtained 50 HCC tissue samples and an equal number of paired adjacent non-tumor tissue samples for qRT-PCR analysis. A TMA containing 313 pairs of HCC tissues and adjacent non-tumor tissues was constructed for IHC testing. HCC differentiation was defined according to the Edmondson-Steiner classification. Micrometastaes were defined as tumors adjacent to the border of the primary tumor that could be observed only under a microscope. All tissue samples were randomly collected at the Eastern Hepatobiliary Surgery Hospital (Shanghai, China) from September 2005 to March 2012 and were stored at −80°C until needed for further use. Two pathologists re-evaluated the tissues independently. The study was approved by the ethics committee of the Eastern Hepatobiliary Surgery Hospital, and written informed consent was obtained from all the study participants, in accordance with the policies of the ethics committee. RFS was defined as the period extending from the date of tumor resection to the date of tumor recurrence detection, the date of death from a non-HCC cause, or date of the last follow-up visit, and OS was defined as the length of time between the date of surgery and either the date of death of the patient or the date of the last follow-up visit. Information that could reveal the identities of the patients was excluded from this report.

### Cell culture

The indicated hepatocellular carcinoma cell lines (SMMC-7721, HCC-LM3, Huh7, HepG2, MHCC-97 L, MHCC-97H) and normal liver cell line (THLE-3) were obtained from the China Center for Type Culture Collection (Wuhan, China) and were authenticated by the provider using DNA-fingerprinting or isoenzyme analysis. All cell lines were maintained in DMEM (HyClone, UT, USA) containing 10% fetal bovine serum and 1% penicillin/streptomycin (GibcoBRL, MD, USA) at 37°C in a humidified atmosphere of 5% CO2.

### RNA extraction, cDNA preparation, and qRT-PCR analysis

Total RNA was extracted from frozen tissues or cells using Trizol reagent (Takara, Dalian, China), according to the manufacturer's instructions. Reverse-transcription was performed using 1–2 μg of total RNA, random primers, and the M-MLV Reverse-transcriptase Kit (Invitrogen, CA, USA), and qRT-PCRwas performed using SYBR Green Master Mix (Takara, China) in a StepOne Plus system (Applied Biosystems, CA, USA). β-actin served as an endogenous control. The qRT-PCR primers are listed in Supplementary Table 2. Relative RNA expression levels were calculated using the comparative Ct method.

### IHC tissue sections and TMA analyses

Paraffin-embedded tissue sections and TMAs underwent IHC analyses. Briefly, the slides were probed with primary antibodies specific for the following proteins CBX6, PCNA, and Ki67, and then the slides were treated with anti-rabbit or anti-mouse horseradish peroxidase-conjugated secondary antibodies (Santa Cruz Biotechnology). Finally, the slides were stained with diaminobenzidine (DAB) colorimetric reagent solution from Dako (Carpinteria, CA, USA) before undergoing hematoxylin counterstaining (Sigma Chemical Co). TMA analysis was performed by scanning the slides with an AperioScanScope GL, and AperioImageScope software (Aperio Technologies, Vista, CA, USA) was used to assess the scanned images by determining the percentages of positively stained cells and staining intensities. CBX6expression in all the clinical samples was quantified, and the tumor CBX1-8 expression level/peri-tumor CBX1-8 expression level ratio was calculated.

### Westernblot analysis

Total cell and tissues lysates were prepared in 1× sodium dodecyl sulfate buffer. Identical quantities of proteins were separated by sodium dodecyl sulfate-polyacrylamide gel electrophoresis and transferred onto nitrocellulose filter membranes. The blots were incubated with antibodies specific for CBX6 (Abcam, CA, USA), S100A9 (Abcam, CA, USA), total-ERK1/2(Abcam, CA, USA), phospho-ERK1/2 (Abcam, CA, USA),total-p38(Abcam, CA, USA), phospho-p38(Abcam, CA, USA), total-p65 (Abcam, CA, USA), phospho-p65 (Abcam, CA, USA), total-p50 (Abcam, CA, USA), phospho-p50 (Abcam, CA, USA), or GAPDH (Abcam, CA, USA), after which they were incubated with IRDye 800-conjugated goat anti-rabbit IgG and IRDye 700-conjugated goat anti-mouse IgG, and the signals were detected using an Odyssey infrared scanner (Li-Cor). GAPDH was used as a loading control for these experiments.

### Animal studies

The animal studies were approved by the Institutional Animal Care and Use Committee of the Second Military Medical University, Shanghai, China. Male athymic BALB/c nude mice (4–5 weeks old) were used for these experiments and received humane care throughout the experimental period. Equal numbers (1 × 10^7^) of transduced HCC-LM3-CBX6i or SMMC-7721-CBX6 cells and the appropriate control cells were simultaneously injected into the bilateral armpits of each mouse. Tumor length (L) and width (W) were measured weekly after injections, and tumor growth curves were plotted using tumor volumes (V = 0.5 × L × W^2^) at each time point. All mice were sacrificed five weeks following injection.

### CCK8 assay and colony-formation assay

Approximately 1000HCC cells were plated in 96-well plates, and cell viability was assessed in 3 replicates from 3 independent experiments by CCK8 (Dojindo, Kumamoto, Japan) every 12 hours. Cell proliferation curves were plotted using the absorbance values at each time point.

For colony-formation assay, the cells were seeded in 6-well plates at a density of 1000 cells per well. After 2 weeks of growth, the surviving colonies were fixed with 4% paraformaldehyde, stained with 0.1% crystal violet, and counted.

### Apoptosis analyses

The cells were pretreated with apoptosis-inducers A (Apopida) and B (Apobid) (1:1,000, Beyotime, China) for 6 h. The cells were subsequently trypsinized, rinsed with PBS, resuspended in 1× binding buffer, stained with annexin V-FITC/PI (BD Bioscience, CA, USA), and analyzed with a flow cytometer (BD Biosciences, CA, USA).

### Enzyme activity assay

Enzyme activity assay (Genmed Scientifics Inc, Shanghai,China) for ERK1/2, p38, p65, and p50 was used to measure the intracellular enzyme activity of the above proteins. Fifty micrograms of protein samples were added to a 96-well-plate, and the corresponding enzyme activity levels were determined using an enzymelabeling instrument at different wavelengths, according to the manufacturer's instructions. The values are presented as a percentage (%) of the control.

### Gene expression profiling and analysis

Total RNA was extracted using TRIzol Reagent (Life Technologies, Carlsbad, CA, USA), according to the manufacturer's instructions, and the RNA integrity number (RIN) was determined using an Agilent Bioanalyzer 2100 (Agilent Technologies, Santa Clara, CA, USA). Acceptable total RNA preparations were purified further using an RNeasy Mini Kit (QIAGEN, GmBH, Germany) and RNase-Free DNase (QIAGEN, GmBH, Germany). Total RNA was amplified and labeled using a Low-input Quick Amp Labeling Kit, One-Color (Agilent Technologies, Santa Clara, CA, USA), according to the manufacturer's instructions, and labeled cRNA was purified using an RNeasy Mini Kit (QIAGEN, GmBH, Germany). Each slide was hybridized with 1.65 μg of Cy3-labeled cRNA in a hybridization oven using a Gene Expression Hybridization Kit (Agilent Technologies, Santa Clara, CA, USA), according to the manufacturer's instructions. After 17 h of hybridization, the slides were washed in staining dishes (Thermo Shandon, Waltham, MA, USA) using a Gene Expression Wash Buffer Kit (Agilent Technologies, Santa Clara, CA, USA), according to the manufacturer's instructions. The slides were scanned with an Agilent Microarray Scanner (Agilent Technologies, Santa Clara, CA, USA).The default settings were used. Data were extracted with Feature Extraction Software 10.7 (Agilent Technologies, Santa Clara, CA, USA) at the Biotechnology Corporation, Shanghai, PR China. The raw data were normalized using the Quantile algorithm in GeneSpring 11.0 (Agilent Technologies, Santa Clara, CA, USA), and we considered genes that were up- or down-regulated by more than 1.2-fold in the independent biological duplicates.

### Statistical analysis

All statistical analyses were performed using SPSS version 17.0 and GraphPad Prism 5.0 software.χ2 test or Fisher's exact test was used for analysis of qualitative variables, and Student's *t*-test or the Mann-Whitney test was performed as appropriate for analysis of quantitative variables. Survival curves were calculated using the Kaplan-Meier method and were compared by a log-rank test. Cox's proportional hazards model was used to identify the independent factors for survival and recurrence, based on variables selected after the univariate analysis. Two-tailed tests were performed to generate *p*-values and *p <* 0.05 was considered statistically significant.

## SUPPLEMENTARY MATERIALS FIGURES AND TABLES


