# Supplementary material for: CBX6 overexpression contributes to tumor progression and is predictive of a poor prognosis in hepatocellular carcinoma
Source: Oncotarget. 2017 Jan 20;8(12):18872–84. doi: 10.18632/oncotarget.14770 (PMC5386654; doi:10.18632/oncotarget.14770)
Supplement: Supplementary file 1 [file oncotarget-08-18872-s001.pdf]

CBX6 overexpression contributes to tumor progression and is predictive of a poor prognosis in hepatocellular carcinoma

Supplementary Materials

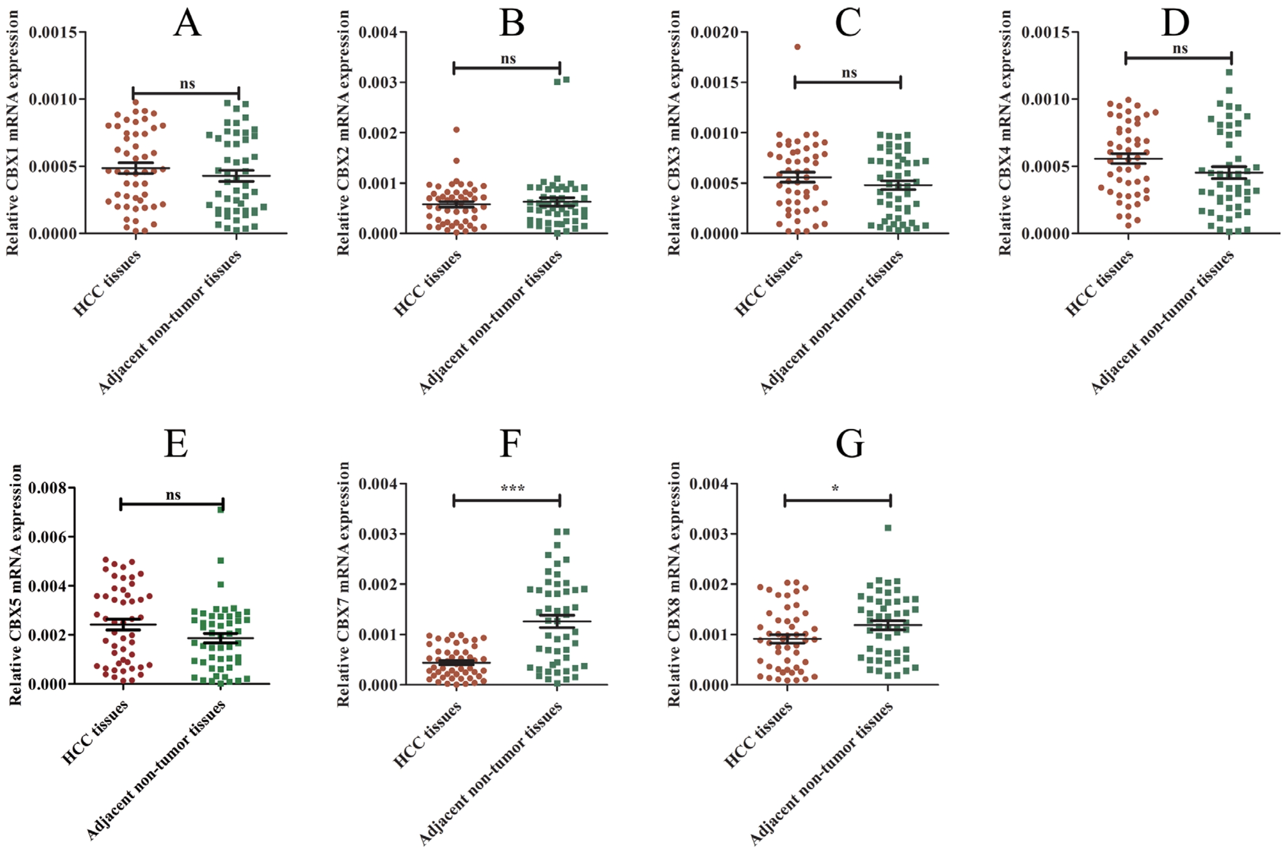

Supplementary Figure 1: CBX family protein expression was measured in HCC samples. (**A–G**) CBX1-5, CBX7 and CBX8 expression in 50 pairs of HCC tissues and matched adjacent non-tumor tissueswas measured via real-time qPCR.

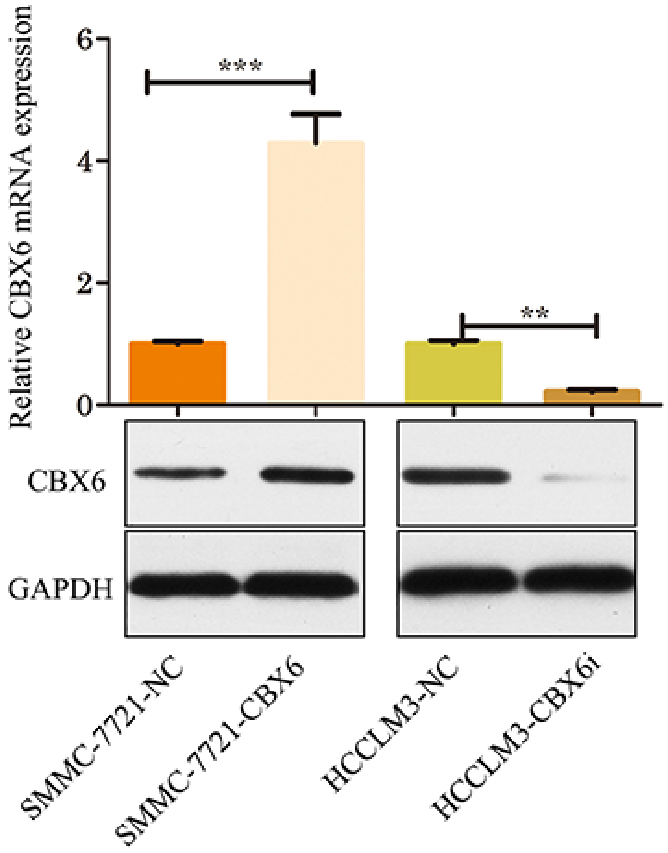

Supplementary Figure 2: CBX6 was stably overexpressed in SMMC-7721 cells and knocked down in LM3 cells. qRT-PCR and Western blotting were performed to assess CBX6 overexpression in SMMC-7721 cells and CBX6 knockdown in LM3 cells (*$p < 0.05$;**$p < 0.01$; ***$p < 0.001$).

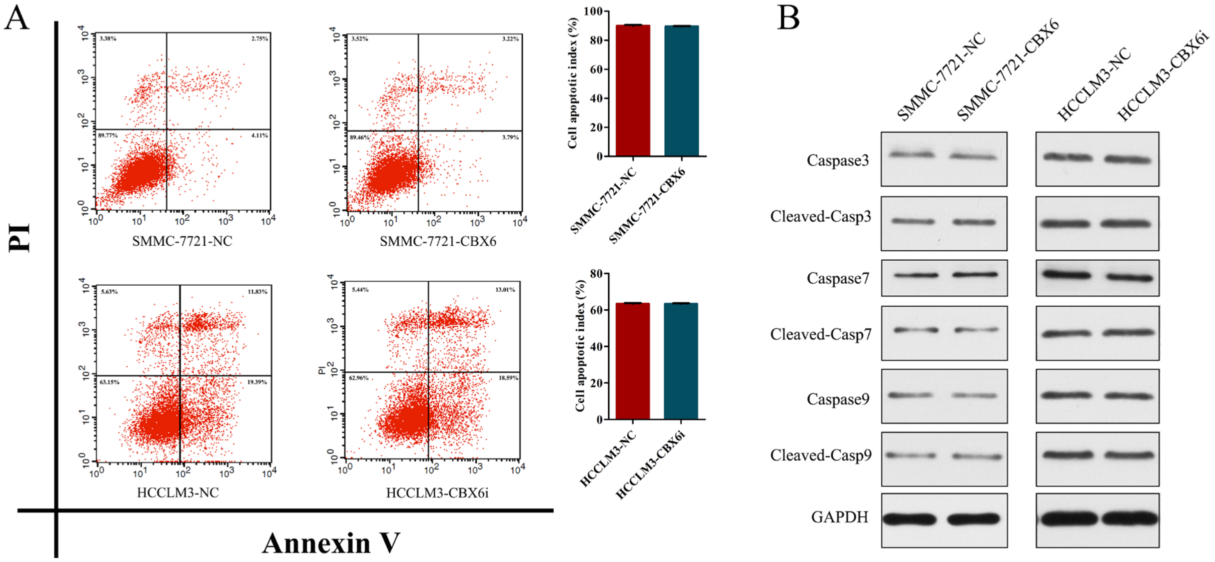

Supplementary Figure 3: Anti-apoptotic effects of PON3 were not observed in HCC cells. (**A**) Flow cytometry analysis of apoptosis using annexin V-FITC and PI staining in CBX6-overexpression and knockdown cells. (**B**) Westernblot analysis of the expression of the apoptosis-related proteins caspase-3, 7, 9 and their respective cleavage fragments in CBX6-overexpression and knockdown cells. GAPDH was used as an internal control.

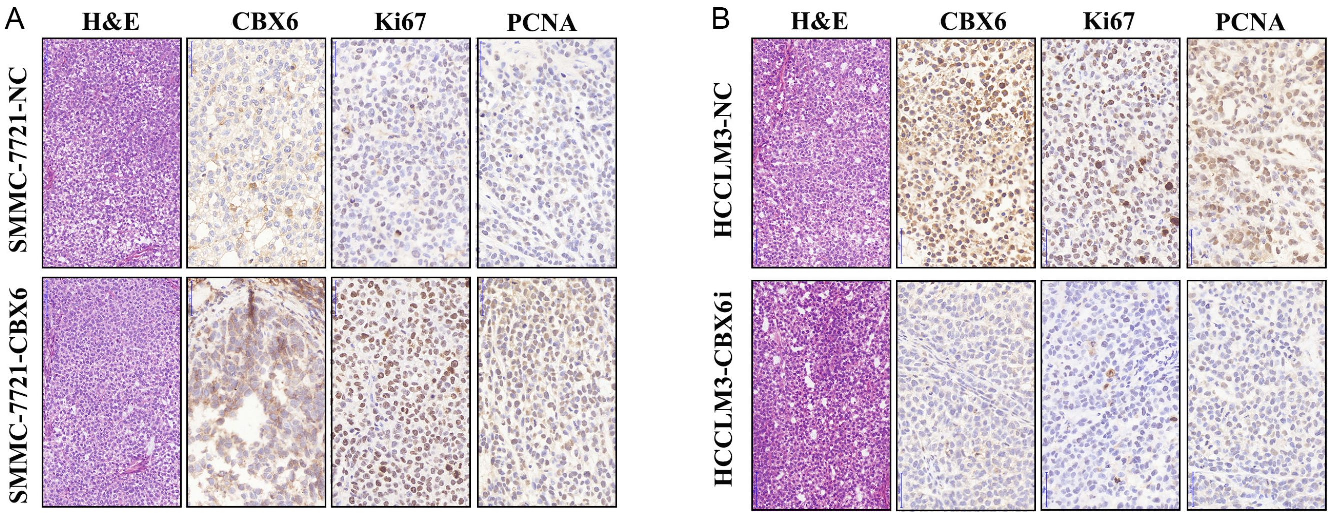

Supplementary Figure 4: CBX6 promotes HCC tumor growth *in vivo*. (**A**) H&E-stained paraffin-embedded sections obtained from the xenografts. IHC staining shows that Ki67 and PCNA expression levels were enhanced in the SMMC-7721-CBX6 group compared with the SMMC-7721-NC group. (**B**) H&E-stained paraffin-embedded sections obtained from the xenografts. IHC staining shows that Ki67 and PCNA expression levels weredecreased in the HCCLM3-CBX6igroup compared with the HCCLM31-NC group.

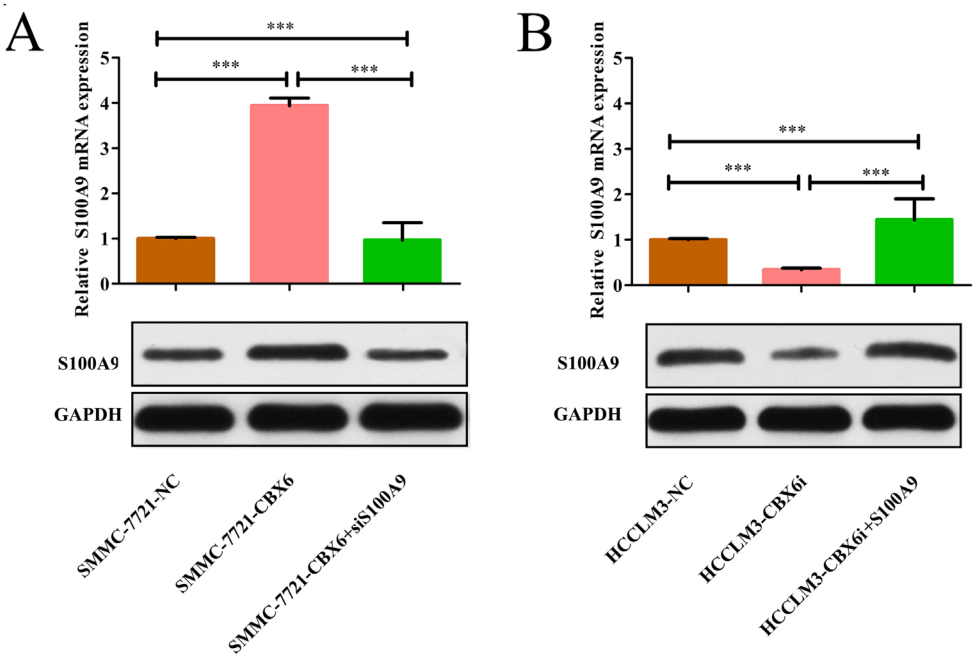

Supplementary Figure 5: S100A9 plays a critical role in mediating CBX6 function. (**A**) S100A9 mRNA and protein levels in SMMC7721 cells overexpressing S100A9 that were transduced with siS100A9 or a control vector were detected by qRT-PCR and westernblotting. (**B**) S100A9 mRNA and protein levels in HCCLM3 CBX6-knockdown cells that were transduced with siS100A9 or a control vector were detected by qRT-PCR and westernblotting (*p < 0.05, **p < 0.01, ***p < 0.001).

Supplemental Table 1: Univariable analysis for recurrence free survival (RFS) and overall survival (OS)

Variable	RFS			OS		
	Hazard ratio	95% CI	*P* value	Hazard ratio	95% CI	*P* value
**Age**, years, ≥ 55 vs. < 55	1.182	0.879–1.591	0.268	1.072	0.790–1.456	0.655
**Sex**, male vs. female	1.139	0.729–1.778	0.568	1.178	0.746–1.860	0.482
**HBsAg**, positive vs. negative	1.990	1.191–3.327	0.009	2.067	1.196–3.572	0.009
**HBeAg**, positive vs. negative	1.633	1.173–2.272	0.004	1.692	1.211–2.364	0.002
**AFP**, μg/L ≥ 20 vs. < 20	1.447	1.059-1.977	0.020	1.363	0.992–1.872	0.056
**Edmondson-Steiner classification**, III-IV vs. I-II	1.578	0.930–2.677	0.091	1.312	0.784–2.196	0.300
**Tumor diameter**, cm, ≥ 5 vs. < 5	1.462	1.085–1.970	0.013	1.442	1.061–1.961	0.019
**Tumor number**, multiple vs. solitary	1.237	0.873–1.751	0.231	1.336	0.937–1.905	0.109
**Vascular invasion**, present vs. absent	1.566	1.152–2.129	0.004	1.337	0.979–1.826	0.068
**CBX6 expression**, high vs. low	1.493	1.115–2.000	0.007	1.626	1.202–2.200	0.002

Abbreviation: HBsAg, hepatitis B surface antigen; HBeAg, hepatitis B e antigen; AFP, alpha-fetoprotein.

Supplementary Table 2: Primer sequences for real-time PCR

CBX1, forward	CTTGCAGAATTGGGTGTGTG
CBX1, reverse	CCTCCCTCCCCCAAATAATA
CBX2, forward	GAGCTCCATCGTGCACTACA
CBX2, reverse	CTTTCTGCGTCCTCACCTTC
CBX3, forward	TTGGCAGTTTAGGACCTGCT
CBX3, reverse	TGTTCTTCCTGGCTTTTGCT
CBX4, forward	GTGAAATGGAGAGGCTGGTC
CBX4, reverse	TCTTCCGATATCCCATCAGC
CBX5, forward	CAGGGTTTCTGGTGTGGTCT
CBX5, reverse	AGCAGAAGTGGGGAGTGCTA
CBX6, forward	AGATGTCACCCTGCTCCAAT
CBX6, reverse	AGCCACCTTCTCGAAATCCT
CBX7, forward	GCTGGTTTTCCCCTTTTCTC
CBX7, reverse	AAGCCTCTCTGAAGGGGAAG
CBX8, forward	GGTCGCAGAAGTACAGCACA
CBX8, reverse	TGGCTCCTCGGCTTTGACAGAGT
S100A9, forward	TCGTAAGTTTTGGCCTTTGC
S100A9, reverse	TGGGTGCCCCAGCTTCACAGA
GAPDH, forward	AGGTGAAGGTCGGAGTCAAC
GAPDH, reverse	CGCTCCTGGAAGATGGTGAT
